# Rare Primary Diffuse Large B-Cell Lymphoma Confined to Bone Marrow: Features and Prognosis

**DOI:** 10.32604/or.2025.063484

**Published:** 2025-07-18

**Authors:** Weiwei Chen, Xiaodie Zhou, Huiyu Li, Yuchen Yang, Lu Lu, Chunyan Zhu, Rong Fang, Xiaoyuan Chu, Shuping Zhou, Qian Sun

**Affiliations:** 1 Department of Medical Oncology, Jinling Hospital, Medical School of Nanjing University, Nanjing, 210000, China; 2 Department of Pathology, Jinling Hospital, Medical School of Nanjing University, Nanjing, 210000, China; 3 Department of Gastroenterology, First Affiliated Hospital, Anhui University of Science & Technology, Huainan, 235000, China

**Keywords:** Primary bone marrow lymphomas (PBMLs), diffuse large B-cell lymphoma (DLBCL), rituximab, chemotherapy, prognosis

## Abstract

**Background:**

Primary bone marrow diffuse large B-cell lymphoma (PBM-DLBCL) represents an uncommon yet clinically aggressive hematologic malignancy. Despite its significant clinical impact, this entity lacks standardized diagnostic criteria in current WHO classifications.

**Methods:**

We performed a retrospective analysis of 55 PBM-DLBCL cases from our institutional database and published literature (2001–2022) to characterize disease features and identify prognostic factors, with particular focus on assessing how different treatment regimens influence therapeutic efficacy and long-term outcomes.

**Results:**

The data suggested a potential link between international prognostic index (IPI) scores and poorer survival, albeit without conclusive statistical evidence (*p* = 0.05). Treatment response emerged as a significant prognostic factor, and patients with complete response (CR) demonstrating superior survival in Cox univariate and multivariate analysis (*p* < 0.001). Intensive therapeutic regimens were associated with improved clinical outcomes compared to conventional therapies. While incorporating rituximab into conventional chemotherapy regimens has demonstrated superior clinical outcomes compared to chemotherapy alone in PBM-DLBCL patients.

**Conclusion:**

Our findings highlight the aggressive nature of PBM-DLBCL and underscore the importance of early recognition, risk stratification, and optimized treatment selection for this rare disease entity.

## 1 Introduction

Primary bone marrow lymphomas (PBMLs) are rare and have a poor prognosis, with high relapse rates and short survival [1,2]. Currently, there is no clear and unified definition or diagnostic criteria for PBMLs in the WHO classification, which may be due to the rarity and variety of the disease. PBMLs are often diagnosed late due to non-specific clinical manifestations, and bone marrow examination is usually required for diagnosis [2,3]. The complexity and difficulties of performing a biopsy on bone marrow, which is usually the only available material, further add to the diagnostic challenge [4]. Several primary bone marrow lymphoid malignancies should be included in the differential diagnosis, such as Hodgkin lymphoma (HL), Burkitt lymphoma (BL), follicular lymphoma (FL), diffuse large B-cell lymphoma (DLBCL), peripheral T-cell lymphoma not otherwise specified (PTCL-NOS), ALK-negative anaplastic large cell lymphoma (ALCL), lymphoplasmacytic lymphoma (LPL), hairy cell leukemia, acute lymphoblastic leukemia (ALL), and chronic lymphocytic leukemia (CLL)/small lymphocytic lymphoma (SLL). Additionally, primary bone lymphoma (PBL), Asian-variant intravascular large B-cell lymphoma (IVLBCL), and T-cell/histiocyte-rich large B-cell lymphoma (THRLBCL) must also be excluded [2,5]. PBML-DLBCL represents the most prevalent histopathological subtype and exhibits distinct clinical manifestations that differentiate it from other lymphomas within its classification. The primary subtypes demonstrate characteristic clinical presentations, most notably cytopenias affecting one or more hematopoietic lineages. Owing to its uncommon occurrence (estimated incidence < 1/1,000,000), PBM-DLBCL suffers from three fundamental knowledge gaps: (i) absence of prospective cohort studies characterizing its natural history, (ii) lack of standardized treatment protocols (with 72% of cases receiving adapted nodal lymphoma regimens per retrospective analyses), and (iii) unidentified outcome predictors—no studies have systematically evaluated prognostic factors specific to PBML pathogenesis.

We present a rare case of primary bone marrow diffuse large B-cell lymphoma (PBM-DLBCL), currently classified as DLBCL NOS (2017 WHO). Unlike conventional DLBCL, PMB-DLBCL exhibits distinct clinicopathological features, including involvement without nodal/extramedullary disease, higher incidence of pancytopenia due to marrow infiltration, subtle presentation without palpable masses, and enriched MYD88 L265P mutations, which necessitate tailored diagnostic and therapeutic approaches. Diagnosis was established through stringent histological assessment of serial bone marrow biopsies. The patient initially responded to R-CHOP (rituximab, cyclophosphamide, epirubicin, vincristine, and prednisone), attaining complete response (CR), but experienced rapid relapse and disease-related mortality within 13 months. Given this poor outcome, we conducted a retrospective analysis of our center’s experience and a literature review to delineate PBM-DLBCL’s distinct clinicopathological features, prognostic determinants, and treatment challenges.

This study addresses the current lack of evidence-based treatment guidelines for the rare PMB-DLBCL subtype, establishing a theoretical foundation and tailored therapeutic approaches based on distinct patient characteristics for future clinical management.

## 2 Patients and Methods

### 2.1 Patient Selection and Data Collection

We analyzed data from 70 cases of PBMLs, including one case from our institution and 69 cases identified through a systematic literature search of PubMed and China National Knowledge Infrastructure (CNKI) between 2001 and 2022. The search strategy employed the terms: [Text Word] “Primary bone marrow lymphoma” OR (“Bone marrow lymphoma” [Text Word]) AND (“Diffuse large B-cell lymphoma” [Text Word]). Cases with hepatic or splenic lymphoma involvement were systematically excluded from the analysis to ensure a pure primary bone marrow DLBCL cohort. To ensure data consistency and reliability, all literature-derived cases were independently reviewed by two investigators. Quality assessment focused on the completeness of clinical information (including diagnostic modality, therapeutic protocol, and survival data). Following rigorous quality control measures that excluded 15 cases due to incomplete survival data or unspecified treatment protocols, our final cohort consisted of 55 evaluable cases that met all predefined inclusion criteria. Detailed patient characteristics are summarized in Table 1.

**Table 1:** Clinical and laboratory characteristics of 55 patients with PBM-DLBCL

Variable	Raw data (%)	Missing data imputaition
Age		
≤60	27 (49.09)	27 (49.09)
>60	28 (50.91)	28 (50.91)
Sex		
Female	25 (45.45)	25 (45.45)
Male	30 (54.55)	30 (54.55)
WBC		
<4 × 10^9^/L	23 (41.82)	26 (47.27)
≥4 × 10^9^/L	25 (45.45)	29 (52.73)
NA	7(12.73)	–
Hb		
<90 g/L	29 (52.73)	32 (58.18)
≥90 g/L	21 (38.18)	23 (41.82)
NA	5(9.09)	–
Platelet		
<75 × 10^9^/L	24 (43.64)	30 (54.54)
≥75 × 10^9^/L	21 (38.18)	25 (45.46)
NA	10 (18.18)	–
LDH		
Normal	3 (5.45)	17 (30.90)
High	38 (69.10)	38 (69.10)
NA	14 (25.45)	–
IPI score		
≤3	24 (43.6)	31 (56.4)
>3	22 (40.0)	24 (43.6)
NA	9 (16.4)	–
B symptoms		
Yes	24 (43.64)	24 (43.64)
No	31 (56.36)	31 (56.36)
CR after treatment		
Yes	35 (63.64)	37 (67.27)
No	18 (32.73)	18 (32.73)
NA	2 (3.63)	–

Note: PBM-DLBCL, Primary bone marrow diffuses large B-cell lymphoma; WBC, white blood cell; Hb, hemoglobin; LDH, lactate dehydrogenase; IPI, international prognostic index; CR, complete response; NA, not reported.

The diagnostic criteria for PBM-DLBCL required: (1) histologically proven bone marrow infiltration; (2) absence of detectable extramedullary involvement (confirmed by physical exam and comprehensive imaging including contrast-enhanced CT and whole-body positron emission tomography/computed tomography (PET/CT)); (3) no focal bone lesions or trabecular destruction on biopsy/PET; (4) exclusion of other leukemic/lymphomatous processes (CLL/SLL, prolymphocytic leukemia (PLL), LPL, hairy cell leukemia (HCL), BL, ALL); (5) no confounding malignancies or life-limiting comorbidities; and (6) no subsequent identification of nodal or visceral lymphoma [5].

We further implemented these exclusion criteria: (1) subjects with coexisting neoplasms or severe comorbidities potentially impacting survival rates, along with (2) unclassifiable B-cell lymphoma cases. Given the rarity of primary bone DLBCL, we included only pathologically and radiologically confirmed case reports from the literature. Cases with incomplete laboratory data were excluded from the risk regression and survival analyses.

We collected clinical data of all patients, including sex, age, peripheral blood indicators at first admission, lactate dehydrogenase (LDH) level, β-2 microglobulin (β2-M) level, international prognostic index (IPI) score, therapeutic interventions, treatment outcomes, and imaging characteristics. Diagnostic evaluation involved tripartite bone marrow assessment (aspiration, biopsy, and cytologic smear analysis). The patients received initial treatment regimens including: R-CHOP (rituximab, cyclophosphamide, doxorubicin, vincristine, prednisone), Hyper-CVAD (cyclophosphamide, vincristine, doxorubicin, dexamethasone) ± rituximab, High-dose methotrexate (HD-MTX), R-P-MITCE-BOM (rituximab, prednisolone, mitoxantrone, cyclophosphamide, etoposide, bleomycin, vincristine, methotrexate), R-Hyper-CVAD, R-ME (rituximab, methylprednisolone, etoposide), R-CHOP followed by autologous stem cell transplantation (ASCT), R-THP-COP (rituximab, pirarubicin, cyclophosphamide, vincristine, prednisone), HD-CHOP (high-dose CHOP), COP/CVP (cyclophosphamide, vincristine/vindesine, prednisone), VACOP-B (etoposide, doxorubicin, cyclophosphamide, vincristine, prednisone, bleomycin), EPOCH (etoposide, prednisone, vincristine, cyclophosphamide, doxorubicin) ± rituximab (R-EPOCH), R2 (rituximab plus lenalidomide). For relapsed/refractory disease, common regimens included: GDP (gemcitabine, dexamethasone, cisplatin), BTK (Bruton Tyrosine Kinase)inhibitors: ibrutinib, Immunomodulatory drugs: thalidomide, pomalidomide, HDAC (Histone Deacetylases) inhibitors: chidamide, mTOR inhibitors: everolimus.

### 2.2 Statistical Analysis

To address missing clinical characteristics in our dataset, we implemented multiple imputation using SPSS 26.0 (IBM Corp., Armonk, NY, USA) with a fully conditional specification algorithm. Treatment outcomes were categorized according to standard lymphoma response criteria, with CR distinguished from non-CR outcomes (partial response [PR], stable disease [SD], or progressive disease). Overall survival (OS) was calculated from the date of histological confirmation to either mortality or the final follow-up. Survival analysis was performed using Kaplan-Meier methodology, with between-group comparisons assessed via log-rank testing. Statistical significance (*p* < 0.05, two-tailed) was determined using R Studio version.

## 3 Results

### 3.1 Patient Characteristics in Our Center

In mid-October 2020, a 63-year-old female initially presented to a local hospital with complaints of pain in both the lower back and left hip, leading to difficulty walking, without any obvious cause. She also reported experiencing fatigue and night sweats but denied having a fever, weight loss, or a medical history of autoimmune or lymphoproliferative diseases.

**Figure 1:**
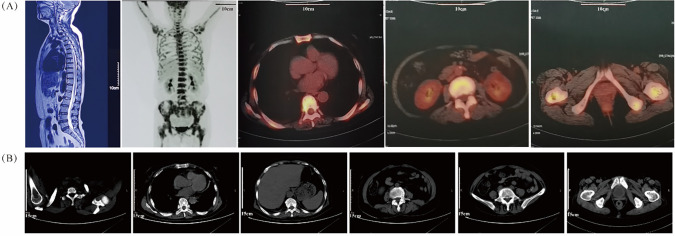
Radiologic results by MRI, PET/CT and CT scans. (**A**) The MRI image (**left panel**) shows multiple abnormal bone signals in the vertebral column. The PET/CT images (**right four panels**) reveal extensive uptake of bone marrow without evidence of involvement in the liver, spleen, or lymph nodes; (**B**) The plain CT scans before treatment do not show any evident bone lesions

Upon physical examination, there were no signs of swelling in the left hip or enlargement of superficial lymph nodes. Magnetic resonance imaging (MRI) revealed multiple abnormal diffuse signals in the pelvis, thoracic and lumbar vertebrae, with local cortical destruction in the femoral heads and bilateral iliac bone. Fluorine-18-fluorodeoxyglucose (18F-FDG) positron emission tomography-computed tomography (PET-CT) showed diffuse increased 18F-FDG uptake in the bone marrow (SUVmax 9.2), without evidence of bone tumors, liver, spleen, or lymph node involvement (Fig. 1A). Subsequent bone marrow smear indicated active proliferation, and flow cytometry revealed no obvious abnormal cells, although most of the laboratory examination results were not available. She was then treated with analgesics and experienced short-term relief of symptoms.

On 01 December 2020, the patient presented to our cancer center with worsening fatigue, night sweats, and pain in both the lower back and left hip. Physical examination revealed new signs of anemia but no jaundice, hepatosplenomegaly, or lymphadenopathy. Plain CT scanning did not show any obvious bone lesions (Fig. 1B). Initial hematologic evaluation demonstrated pancytopenia with leukopenia (WBC 2.1 × 10^9^/L; reference 4.0–10.0 × 10^9^/L), neutropenia (1.40 × 10^9^/L; reference 2.04–7.5 × 10^9^/L), and severe anemia (Hb 68 g/L; reference 110–150 g/L) with erythrocytopenia (RBC 2.40 × 10^12^/L; reference 3.5–5.0 × 10^12^/L). Thrombocytes remained normal (218 × 10^9^/L; reference 100–300 × 10^9^/L). Marked inflammatory activity was evidenced by elevated CRP (103.4 mg/L; reference <8.0 mg/L) and ESR (151 mm/h; reference 0–20 mm/h). Metabolic disturbances included increased LDH (329 U/L; reference 120–246 U/L) and ALP (242 U/L; reference 45–129 U/L), with marginally raised β2-microglobulin (1.6 mg/L; reference 0.9–2.7 mg/L). Immunologic studies revealed monoclonal IgA-λ paraproteinemia without Bence-Jones proteinuria.

**Figure 2:**
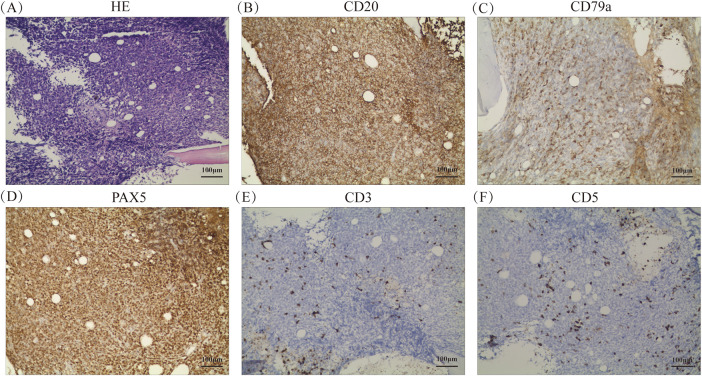

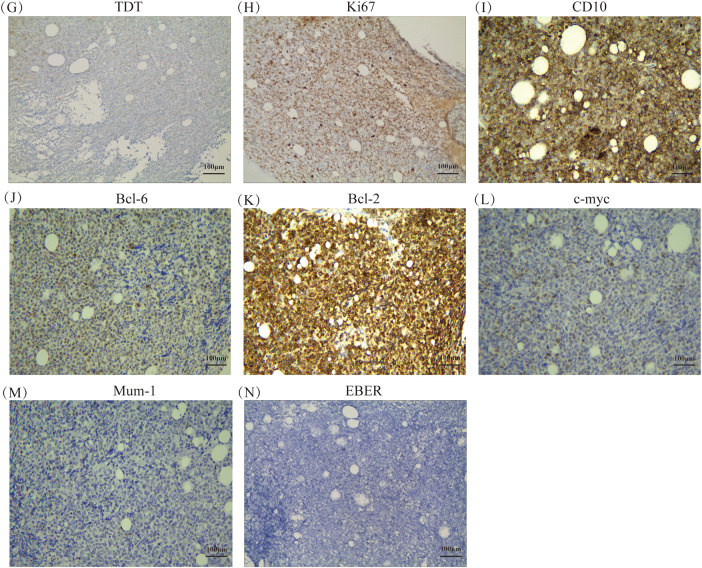
Histopathological and molecular features of bone marrow biopsy (**A**) Hematoxylin and eosin staining at ×200 magnification, respectively. (**B–M**) Immunohistochemical staining results for CD20, CD79a, PAX5, CD3, CD5, TDT, Ki67, CD10, BCL-6, BCL-2, C-MYC, and MUM-1, respectively, at ×200 magnification. (**N**) *In situ* hybridization result for EBER at ×200 magnification

**Figure 3:**
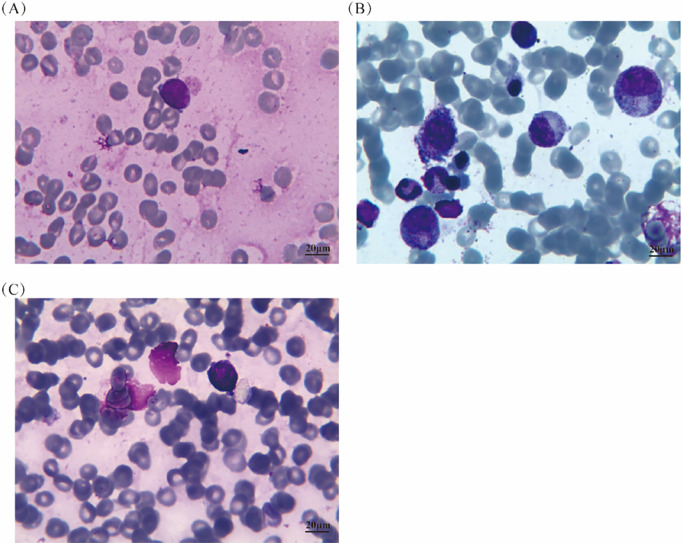
Bone marrow aspirate results at different time points before and after treatment. (**A**) Before treatment, the bone marrow aspirate showed approximately 5.5% of unclassified medium-sized round-like cells with distinct characteristics; (**B**) After two cycles of treatment, the bone marrow cytology returned to normal; (**C**) In the last observation, the bone marrow cytology showed 8% of lymphoma cells

Histopathology of the BM confirmed the diagnosis of DLBCL, which is one of the lymphohematopoietic malignancies (Fig. 2A). Immunohistochemical (IHC) studies of the bone trabeculae showed the intact structure, and the tumor cells exhibited strong diffuse positivity for CD20, CD79a, Pax-5, CD10, and BCL-2. They also showed scattered positivity for CD3, CD5and BCL-6, and negative staining for TDT and MUM-1. The proliferation index, as indicated by Ki-67, was 60%, and C-MYC showed 30% positivity (Fig. 2B–M). The absence of EBER expression by *in situ* hybridization effectively rules out Epstein-Barr virus involvement in the observed lymphoproliferative process (Fig. 2N). To further investigate the pathology, an initial bone marrow (BM) biopsy was performed from the right anterior iliac crest. The results showed a large area of neoplastic necrosis and residual cell shadow among the bone tissues, but without trabecular destruction. Subsequently, a left iliac BM aspiration and trephine biopsy were performed. Microscopic examination of the bone marrow aspirate demonstrated 5.5% atypical mononuclear cells exhibiting: intermediate cell size with rounded morphology; increased nuclear-to-cytoplasmic ratio; sparsely basophilic cytoplasm; prominent nucleolar features; irregular membrane projections at cellular peripheries. These unclassifiable neoplastic-appearing cells raised suspicion for hematopoietic malignancy (Fig. 3A). However, chromosomal karyotype analysis was unsuccessful due to an insufficient quantity of BM sample. Histopathology of the BM confirmed the diagnosis of DLBCL, which is one of the lymphohematopoietic malignancies. Based on these findings, the patient was diagnosed with stage IVB PBM-DLBCL with IPI score of 3. Chemoimmunotherapy commenced on December 11, 2020 using the R-CHOP protocol (rituximab 375 mg/m^2^ day 0; cyclophosphamide 750 mg/m^2^, epirubicin 50 mg/m^2^, and vincristine 1.4 mg/m^2^ day 1; with oral prednisone 100 mg days 1–5), repeated every 21 days for six complete cycles. After two cycles, her symptoms of fatigue, night sweats, and pain disappeared. Additionally, the disease was evaluated as a partial response (PR) based on normal bone marrow cytology without the presence of residual tumor (Fig. 3B), plain CT scan showing no bone lesions, and laboratory tests indicating leukopenia (2.3 × 10^9^/L WBC) with mild anemia (hemoglobin 93 g/L, RBC 3.19 × 10^12^/L) but normal thrombocyte levels (245 × 10^9^/L). Inflammatory and metabolic parameters were unremarkable, including ESR (13 mm/h), LDH (165 U/L), and β2-microglobulin (1.2 mg/L).After completing 6 cycles, the patient achieved a CR with normal bone marrow cytology, CT scan showing no bone lesions, and laboratory tests indicating a WBC count of 2.7 × 10^9^/L, RBC count of 3.6 × 10^12^/L, Hb level of 103 g/L, platelet count of 249 × 10^9^/L, ESR of 12 mm/h, LDH level of 162 U/L, ALP level of 66 U/L, and β2-M level of 1.6 mg/L. The patient was recommended to undergo PET-CT and serum immunofixation electrophoresis, but she refused. In addition, intrathecal methotrexate (MTX) for central nervous system (CNS) prophylaxis therapy was not performed. Subsequently, the patient received a maintenance therapy with thalidomide (100 mg, once daily) until she discontinued it herself in June 2021.

Unfortunately, in September 2021, the patient experienced a relapse of PBM-DLBCL, with bone marrow cytology showing 8% of lymphoma cells (Fig. 3C), CT scan revealing no bone lesions, and laboratory tests indicating a WBC count of 2.2 × 10^9^/L, RBC count of 3.31 × 10^12^/L, Hb level of 92 g/L, platelet count of 154 × 10^9^/L, ESR of 80 mm/h, LDH level of 396 U/L, ALP level of 275 U/L, and β2-M level of 1.7 mg/L. She received one cycle of chemotherapy with gemcitabine (1 g, days 1 and 8), cisplatin (30 mg, days 1–3), and dexamethasone (20 mg, days 1–3). However, the use of gemcitabine on day 8 was withdrawn due to severe thrombocytopenia and anemia. After receiving symptomatic treatment and supportive care, one cycle of lenalidomide (25 mg daily, days 1–21, every 4 weeks) was administered, but it was discontinued due to severe thrombocytopenia. Unfortunately, the patient eventually succumbed to primary bone marrow DLBCL in mid-November 2021.

### 3.2 Literature Review and Statistical Analysis

Our analysis incorporated 55 histologically confirmed PBM-DLBCL cases, demonstrating a slight male predominance (54.55%) with median age 60 years (ranges 18–79) [2,6–32]. Clinically, most patients presented with constitutional B symptoms and high-risk features including: advanced IPI scores (40.00% with score > 3); significant cytopenias (anemia grades 2–4 in 52.73%, thrombocytopenia 43.64%); characteristic metabolic disturbances (elevated LDH in 69.1%). Therapeutic responses were encouraging, with 50% achieving CR following first-line treatment (Table 1).

**Figure 4:**
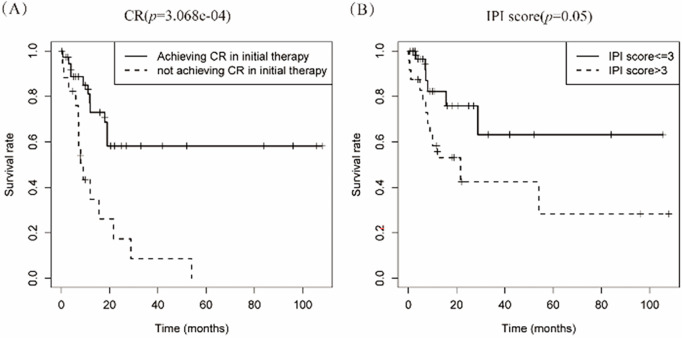
Kaplan-meier curve analyses on the association between survival rate and various factors. (**A**) The curve analysis demonstrates that achieving complete response (CR) in initial therapy is significantly associated with survival rate; (**B**) International Prognostic Index (IPI) score tend to have an impact on prognosis in patients with PBM-DLBCL

Univariate survival analysis of 55 evaluable cases identified treatment-induced CR as the sole statistically significant prognostic variable for PBM-DLBCL (hazard ratio [HR] 3.91, 95% confidence interval [CI] 1.760–8.691; *p* < 0.001). In the multivariate analysis, treatment-induced CR remained the only independent prognostic factor achieving statistical significance (*p* < 0.001), confirming its critical role in determining clinical outcomes for PBM-DLBCL patients (Table 2). Kaplan-Meier curve analysis showed that achieving CR in initial therapy was significantly associated with a better survival rate (*p* = 3.068e−04). Our analysis revealed a marginal association between elevated IPI scores and adverse outcomes in PBM-DLBCL (*p* = 0.05, Fig. 4), suggesting potential but unconfirmed prognostic utility in this rare subtype.

**Table 2:** Cox univariate and multivariate analysis for overall survival of PBM-DLBCL

Variable	Univariate analysis			Multivariate analysis		
	*p*-Value	HR	95% CI	*p*-Value	HR	95% CI
Age	0.913	0.956	0.425–2.147	0.302	0.572	0.198–1.651
Sex	0.326	1.488	0.673–3.288	0.514	1.385	0.520–3.690
WBC	0.173	1.778	0.777–4.070	0.097	2.209	0.867–5.628
Hb	0.682	1.179	0.536–2.594	0.930	0.958	0.363–2.526
Platelet	0.706	1.164	0.529–2.559	0.469	0.698	0.263–1.850
LDH	0.990	1.006	0.399–2.536	0.264	0.502	0.150–1.683
IPI score	0.379	1.175	0.821–1.681	0.442	1.185	0.768–1.830
B symptoms	0.874	1.066	0.483–2.354	0.385	1.488	0.607–3.645
CR after treatment	<0.001	3.911	1.760–8.691	<0.001	6.107	2.348–15.888

Note: PBM-DLBCL, Primary bone marrow diffuse large B-cell lymphoma; WBC, white blood cell; Hb, hemoglobin; LDH, lactate dehydrogenase; IPI, international prognostic index; CR, complete response; HR, Hazard Ratio; CI, confidence interval.

**Figure 5:**
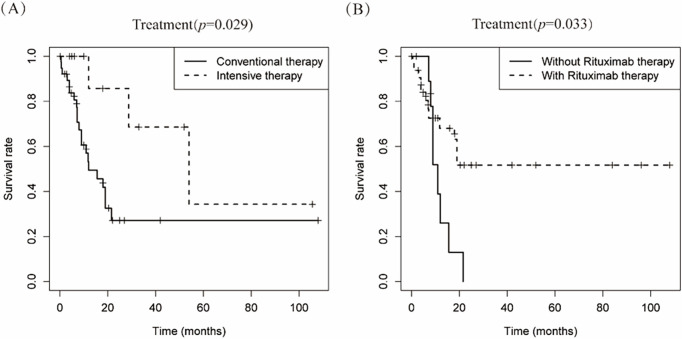
Depicts the cumulative survival curve of patients with PBML-DLBCL who underwent systemic therapy. Subfigures (**A**) illustrate the curves for different treatment approaches, including conventional therapy or intensive therapy and (**B**) the use of rituximab in the conventional therapy group, respectively

Therapeutic approaches were categorized into conventional regimens (R-CHOP/R-CHOP-like/CHOP-like; *n* = 42) vs. intensive protocols (HVPERCAVD ± R/EPOCH ± R/ALL/HD-CHOP/VACOPB; *n* = 12, Table 3). Comparative survival analysis demonstrated superior OS outcomes with intensive treatment strategies (*p* = 0.029). Within the conventional therapy group, rituximab incorporation significantly influenced prognosis (*p* = 0.033, Fig. 5).

**Table 3:** The therapeutic outcomes of 55 patients receiving systemic treatment

No.	Treatment	Outcomes	Reference
1	HEPERCAVD-intrathecal with MTX	CR	[6]
2	R-CHOP	CR	[7]
3	R-P-MITCE-BOM-intrathecal with MTX	CR	[7]
4	CHOP-intrathecal with MTX	no CR	[7]
5	RCHOP-ASCT	CR	[8]
6	R-CHOP	CR	[9]
7	CHOP	PD	[10]
8	R-CHOP	CR	[11]
9	HD-CHOP	PR	[11]
10	CHOP	PD	[11]
11	COP	PR	[11]
12	R-CHOP	CR	[11]
13	R-CHOP	PD	[11]
14	R-CHOP	PD	[11]
15	ALL	PD	[11]
16	VACOPB	CR	[11]
17	R-CHOP	CR	[11]
18	CHOP	PD	[11]
19	R-CHOP	PD	[11]
20	CHOP	PD	[11]
21	R-CHOP	CR	[11]
22	CHOEP	PR	[11]
23	R-CHOP-ASCT	CR	[12]
24	R-CHOP	CR	[13]
25	R-CHOP	CR	[14]
26	R-THP-COP	CR	[15]
27	RCVP	PR	[16]
28	R-CHOP	NA	[16]
29	CVP	CR	[16]
30	CHOP	CR	[16]
31	R-CHOP	CR	[17]
32	REPOCH	CR	[17]
33	R-CHOP	CR	[18]
34	R-CHOP	CR	[19]
35	NO	not CR	[19]
36	CHOP	CR	[19]
37	R-CHOP	NA	[20]
38	EPOCH	PR	[21]
39	R-CHOP-intrathecal with MTX	CR	[22]
40	R-CHOP	CR	[23]
41	R-CHOP	CR	[24]
42	R-CHOP	CR	[25]
43	R-CHOP-intrathecal with MTX	CR	[26]
44	R-CHOP	CR	[27]
45	EPOCH	CR	[27]
46	REPOCH	CR	[28]
47	Rituximab + methylprednisolone + etoposide	PD	[2]
48	Etoposide + dexamethasone + cyclosporine	CR	[2]
49	R-CHOP	CR	[2]
50	Rituximab + methylprednisolone + etoposide	PR	[2]
51	R-Hyper-CVAD-intrathecal with MTX	CR	[29]
52	R	not CR	[30]
53	R-CHOP	CR	[31]
54	R-CHOP–HD-MTX	CR	[32]
55	R-CHOP	CR	our center

Note: CR, Complete Response; PR, Partial Response; SD, Stable Disease; PD, Progressive Disease.

## 4 Discussion

PBM-DLBCL, an ultra-rare and clinically aggressive lymphoma, manifests with nonspecific systemic symptoms (e.g., B symptoms, cytopenias), often delaying diagnosis. To date, its molecular drivers and optimal therapies remain poorly defined due to reliance on low-level evidence (case series/small retrospectives). Crucially, no prospective trials or consensus guidelines exist for this disease, underscoring an unmet need in precision oncology.

In this case, we report on a female patient who initially complained of pain, fatigue, and night sweats, but did not show any remarkable or specific findings in lab tests and bone marrow cytology. However, MRI and PET-CT scans revealed increased metabolic activity in the pelvis and vertebral columns. Six weeks later, the final diagnosis of PBM-DLBCL was confirmed through two additional bone marrow aspirations and biopsies, highlighting the importance of repeat or multiple bone marrow biopsies, as suggested in previous reports [5]. IHC results indicated that the lymphoma cells strongly and diffusely expressed CD10 and BCL-2, with scattered positivity for BCL-6 and CD5, 30% positivity for C-MYC, and negativity for MUM-1. Previous studies have shown that the non-germinal center B-cell-like (non-GCB) type is more common in PBM-DLBCL than in patients with secondary bone marrow involvement, although the difference may not be significant [5,33]. Based on the Hans classification, the present case tended to be classified as PBM-DLBCL of the GCB type, with possible triple-hit expressions of BCL-2, BCL-6, and C-MYC. However, chromosomal karyotype analysis was unsuccessful due to insufficient BM sample quantity, leading to uncertainty about gene rearrangement or translocation.

While comprehensive epidemiological data remain limited, current studies estimate that PBM-DLBCL accounts for 2.4% of DLBCL presentations, substantially less common than secondary bone marrow infiltration which occurs in 11%–20% of DLBCL cases [33]. Although there is no clearly unified criteria for defining primary bone marrow lymphoma, many reports have demonstrated the major clinical features of these malignancies, including fever (especially of unknown origin), cytopenia, elevated LDH levels, and worse outcomes. Compared to patients with secondary bone marrow involvement, PBM-DLBCL patients were found to have more cytopenia (unilineage, bilineage or trilineage), cytogenetic aberrations, hemophagocytic lymphohistiocytosis (HLH), and atypical lymphocytes in peripheral blood [5,33]. Notably, cytogenetic aberrations and leukocytosis demonstrated prognostic significance exclusively in secondary involvement cases, highlighting the need to distinguish these entities despite DLBCL’s rare primary marrow presentation. The prognosis for PBM-DLBCL is usually very poor, with a 1-year survival rate of about 33%, significantly lower than that of DLBCL patients with secondary bone marrow involvement (49%, *p* = 0.022) [33]. In some retrospective studies, the median survival time was reported to be only half a month, and most patients with PBM-LBCL died within one month. While CD5-positive DLBCL is broadly associated with aggressive features (e.g., non-GCB phenotype, extranodal/CNS spread, and rituximab resistance) [5], its prognostic impact in PBM-DLBCL remains unvalidated due to limited cases. In this patient, scattered CD5 positivity was observed, but its contribution to the rapid relapse and short survival (despite initial R-CHOP response) cannot be conclusively determined. To date, rituximab-containing chemotherapy remains the sole established prognostic factor for both PBM-DLBCL and secondary bone marrow involvement [33].

The updated WHO classification mandates molecular subtyping of DLBCL into cell-of-origin categories, distinguishing tumors by gene expression profiles (GEP) resembling either germinal center B cells (GCB subtype) or activated B cells (ABC subtype) [34]. The consistently worse prognosis of ABC-DLBCL patients, observed initially with CHOP and subsequently with R-CHOP regimens, underscores the critical influence of molecular subtype on therapeutic response. The GEP-based Bayesian classifier developed by Wright et al. assigned linear predictor scores (LPS) to DLBCL cases, yet failed to classify 10%–15% of tumors due to ambiguous ABC/GCB signatures, highlighting biological heterogeneity. Recently, a novel multiplatform genomic predictor classification of DLBCL has been identified using next-generation sequencing (NGS) and transcriptome analyses on large-sized biopsy samples [35,36]. This classification defines six genetic subtypes in DLBCL, including BN2 (based on BCL6 fusions and NOTCH2 mutations), A53(Biallelic TP53 inactivation), EZB (based on EZH2 mutations and BCL2 translocations, be molecularly partitioned into EZB-DZ+ (BCL2 translocation + EZH2 mutation) and EZB-DZ− (wild-type for both)), ST2 (TET2/SGK1,SOCS1/SGK1), MCD (based on the co-occurrence of MYD88L265P and CD79B mutations), and N1 (based on NOTCH1 mutations). The genetic landscape of DLBCL predicts clinical outcomes: EZB-DZ+ and MCD/N1 subgroups exhibit poor prognosis, while ST2 mirrors favorable GCB-DLBCL outcomes. BN2 shows neutral survival, and A53’s prognostic relevance remains uncertain despite TP53-associated risks [1,3,34].

The absence of prospective clinical trials specifically evaluating PBM-DLBCL has forced clinicians to adapt systemic DLBCL treatment paradigms, despite the biological and clinical distinctions between these disease entities. The treatment of PBM-DLBCL remains challenging, despite the consensus on rituximab-containing chemotherapy as the first-line standard of care and improved next-line options. This is mainly due to the profound molecular heterogeneity [2,30–33]. The first-line therapy with R-CHOP achieves cure rates of about 60%–70% across all DLBCL patients, but the 1-year survival rate in PBM-DLBCL is only about 33% [33,34,37,38]. Increasing the intensity of treatment by using 8 cycles of R-CHOP instead of 6 has been reported to result in an inferior outcome [37]. Multiple randomized controlled trials have consistently shown that rituximab-containing regimens achieve significantly better survival rates in DLBCLs. The therapeutic advantage primarily stems from rituximab’s ability to mediate antibody-dependent cellular cytotoxicity (ADCC) and complement-dependent cytotoxicity (CDC), while also potentiating chemosensitivity through synergistic mechanisms with cytotoxic agents. Furthermore, clinical trials with intensified chemotherapy regimens such as R-CHOEP, DA-R-EPOCH, and R-ACVBP have failed to further improve the prognosis in systemic DLBCL [34–36]. Other intensive regimens, including HVPERCAVD, EPOCH, ALL, HD-CHOP, and VACOPB, have shown some benefit but also come with greater risks in PBMLs compared to the CHOP or CHOP-like regimens as observed in our study, may stem from this malignancy’s uniquely aggressive biology, which compromises patient tolerance to dose-intensified therapies [2]. The intensive treatment regimens examined in this study—Hyper-CVAD ± R, R-P-MICE-BOM, VACOP-B, R-CHOP with sequential high-dose methotrexate, and dose-adjusted EPOCH ± R—demonstrate optimal therapeutic efficacy in carefully selected PMB-DLBCL patients. Current clinical evidence identifies three key patient selection criteria for these intensive protocols: (1) high-risk disease status (IPI score ≥ 3), (2) age < 60 years with preserved hematologic function, and (3) adequate organ performance status. This specific patient population exhibits enhanced treatment tolerance, enabling maintenance of optimal dose intensity without significant treatment delays or dose reductions. From a mechanistic perspective, these intensive regimens provide dual therapeutic advantages: effective suppression of tumor cell repopulation and overcoming of tumor DNA repair mechanisms.

However, intensive chemotherapy may not be necessary or appropriate for all patients. Crucially, molecular profiling studies have revealed particular efficacy in distinct genetic subtypes, including the MCD (MYD88L265P/CD79B-mutated), BN2 (BCL6-rearranged with NOTCH2 mutations), and double-hit lymphoma subgroups, suggesting these intensive approaches may effectively address the therapeutic challenges posed by these biologically aggressive variants. These findings underscore the importance of comprehensive patient evaluation incorporating both clinical and molecular characteristics when considering intensive treatment strategies. The PHOENIX trial demonstrated age- and subtype-dependent efficacy of ibrutinib + R-CHOP in non-GCB DLBCL [39]. While the overall trial was negative, significant benefit was observed in patients < 60 years (improved EFS/PFS/OS) and those with MCD/N1 subtypes increased dependence on nuclear factor-kappa B (NF-kB) signaling (100% 3-year EFS) [1,3,35,36]. Conversely, older patients had worse outcomes, likely due to treatment-limiting toxicity.Antibody-drug conjugates (ADCs) represent a promising therapeutic approach for lymphoma. Loncastuximab tesirine (LT), a CD19-targeting ADC with DNA-damaging payload SG3199, demonstrated clinical activity in relapsed/refractory DLBCL (LOTIS-2: CR 24.8%, median PFS 4.9 months) [40]. Notably, complete responders achieved durable responses (2-year PFS 72.5%, OS 68.2%) [41]. Early-phase trials show enhanced efficacy in combinations: LOTIS-3 (with ibrutinib) reported ORR 57.1%/CR 34.3% (prematurely terminated) [41], while ongoing LOTIS-5 (with rituximab) shows promising preliminary results (ORR 80%/CR 50%) [42]. In frontline therapy, the phase III POLARIX trial demonstrated superior efficacy of Pola-R-CHP (polatuzumab plus R-CHP) over R-CHOP, with 2-year PFS rates of 76.7% vs. 70.2% (*p* = 0.02) [43]. The addition of lenalidomide to R-CHOP (R2-CHOP) showed subtype-dependent efficacy across trials. In the ROBUST study, significant benefit was observed specifically in high-risk (IPI 3–5) ABC-type DLBCL [44]. Conversely, the E1412 trial demonstrated improved PFS across all molecular subtypes (GCB and non-GCB), suggesting broader activity in unselected populations [45,46]. Additionally, autologous stem cell transplantation (Auto-SCT) and CAR T-cell therapies show promise in redefining the treatment landscape of PBMLs but require further confirmation [2,34,44,45].

## 5 Conclusion

In conclusion, PBM-DLBCL is an aggressive yet treatable malignancy. Early diagnosis through BM biopsy and prompt initiation of chemotherapy plus rituximab are crucial. It should be noted that our study was conducted on a relatively small scale, therefore, it remains unclear whether patients achieving complete remission after systemic therapy require intrathecal methotrexate or maintenance therapy such as BTK inhibitors.

Our comprehensive analysis of PMB-DLBCL research progress identified critical knowledge gaps. However, inherent limitations of retrospective studies, including small sample sizes and incomplete clinical data, may introduce potential biases in our conclusions. These methodological constraints highlight the necessity for multicenter, large-scale prospective studies to establish optimal therapeutic strategies for PMB-DLBCL.

## Data Availability

All data generated or analyzed during this study are included in this published article.

## References

[1] Hilton LK, Scott DW, Morin RD. Biological heterogeneity in diffuse large B-cell lymphoma. Semin Hematol. 2023;60(5):267–76. doi:10.1053/j.seminhematol.2023.11.006; 38151380

[2] Wang G, Chang Y, Wu X, Li X, Li L, Zhang L, et al. Clinical features and prognostic factors of primary bone marrow lymphoma. Cancer Manag Res. 2019;29(11):2553–63 doi: 10.2147/cmar.s187522; 31015766 PMC6446986

[3] Sehn LH, Salles G. Diffuse large B-cell lymphoma. N Engl J Med. 2021;384(9):842–58. doi:10.1056/nejmra2027612; 33657296 PMC8377611

[4] Zhao J, Wang WJ, Wang JC, Du HZ, Guan JH. Role of bone marrow cell morphology combined with immunohistochemistry examinations in the diagnosis of patients with primary bone marrow lymphoma. Zhongguo Shi Yan Xue Ye Xue Za Zhi. 2021;29(6):1807–11. doi:10.19746/j.cnki.issn.1009-2137.2021.06.020; 34893115

[5] Zamò A, Johnston P, Attygalle AD, Laurent C, Arber DA, Fend F. Aggressive B-cell lymphomas with a primary bone marrow presentation. Histopathology. 2020;77(3):369–79. doi:10.1111/his.14124; 32324290

[6] Matthies A, Schuster SJ, Alavi A. Staging and monitoring response to treatment in primary non-Hodgkin’s lymphoma of bone marrow using ^18^F-fluorodeoxyglucose positron emission tomography. Clin Lymphoma. 2001;1(4):303–6. doi:10.3816/clm.2001.n.006; 11707846

[7] Alvares CL, Matutes E, Scully MA, Swansbury J, Min T, Gruszka-Westwood AM, et al. Isolated bone marrow involvement in diffuse large B cell lymphoma: a report of three cases with review of morphological, immunophenotypic and cytogenetic findings. Leuk Lymphoma. 2004;45(4):769–75. doi:10.1080/10428190310001625746; 15160954

[8] Hishizawa M, Okamoto K, Chonabayashi K, Kaneko H, Watanabe M, Tsudo M. Primary large B-cell lymphoma of the bone marrow. Br J Haematol. 2007;136(3):351. doi:10.1111/j.1365-2141.2006.06336.x; 17233843

[9] Níáinle F, Hamnvik OP, Gulmann C, Bermingham C, Kelly J, Mc Evoy P, et al. Diffuse large B-cell lymphoma with isolated bone marrow involvement presenting with secondary cold agglutinin disease. Int J Lab Hematol. 2008;30(5):444–5. doi:10.1111/j.1751-553x.2007.00977.x; 18205841

[10] Chang H, Hung YS, Lin TL, Wang PN, Kuo MC, Tang TC, et al. Primary bone marrow diffuse large B cell lymphoma: a case series and review. Ann Hematol. 2011;90(7):791–6. doi:10.1007/s00277-010-1129-4; 21181164

[11] Martinez A, Ponzoni M, Agostinelli C, Hebeda KM, Matutes E, Peccatori J, et al. International Extranodal Lymphoma Study Group. Primary bone marrow lymphoma: an uncommon extranodal presentation of aggressive non-hodgkin lymphomas. Am J Surg Pathol. 2012;36(2):296–304. doi:10.1097/pas.0b013e31823ea106. 22251943

[12] Kazama H, Teramura M, Yoshinaga K, Masuda A, Motoji T. Long-term remission of primary bone marrow diffuse large B-cell lymphoma treated with high-dose chemotherapy rescued by *in vivo* rituximab-purged autologous stem cells. Case Rep Med. 2012;2012(2):957063. doi:10.1155/2012/957063; 23118770 PMC3480674

[13] Niscola P, Palombi M, Fratoni S, Perrotti A, de Fabritiis P. Primary diffuse large B-cell lymphoma of the bone marrow in a frail and elderly patient successfully treated with rituximab, cyclophosphamide, doxorubicin, vincristine, and prednisone. Blood Res. 2013;48(4):296–7. doi:10.5045/br.2013.48.4.296; 24466557 PMC3894391

[14] Lapa C, Knott M, Rasche L, Herrmann K, Buck AK, Rosenwald A. Primary bone marrow diffuse large B-cell lymphoma affecting distal parts of the legs as a cause of persisting B symptoms. Eur J Haematol. 2014;93(6):545–6. doi:10.1111/ejh.12305; 24612353

[15] Yamashita T, Ishida M, Moro H, Yumoto H, Uchibayashi S, Yoshii M, et al. Primary bone marrow diffuse large B-cell lymphoma accompanying cold agglutinin disease: a case report with review of the literature. Oncol Lett. 2014;7(1):79–81. doi:10.3892/ol.2013.1695; 24348825 PMC3861573

[16] Bhagat P, Sachdeva MU, Sharma P, Naseem S, Ahluwalia J, Das R, et al. Primary bone marrow lymphoma is a rare neoplasm with poor outcome: case series from single tertiary care centre and review of literature. Hematol Oncol. 2016;34(1):42–8. doi:10.1002/hon.2178; 25407700

[17] Liu H, Yi S, Liu E, Li Z, Zhang H, Ru K, et al. Primary bone marrow diffuse large B cell lymphoma: three case reports and literature review. Zhonghua Xue Ye Xue Za Zhi. 2014;35(10):914–7. doi:10.3760/cma.j.issn.0253-2727.2014.10.009; 25339329

[18] Kosugi S, Watanabe M, Hoshikawa M. Primary bone marrow lymphoma presenting with cold-type autoimmune hemolytic anemia. Indian J Hematol Blood Transfus. 2014;30(Suppl 1):271–4. doi:10.1007/s12288-014-0356-6; 25332595 PMC4192235

[19] Gao J, Chen BL, Tian M, Yue XY, Tang JQ, Tian LT, et al. Primary bone marrow lymphoma: a report of three cases and literature review. J Clin Hematol. 2015;28(11):983–4.

[20] Hu Y, Chen SL, Huang ZX, Gao W, An N. Case report diffuse large B-cell lymphoma in the primary bone marrow. Genet Mol Res. 2015;14(2):6247–50. doi:10.4238/2015.june.9.10; 26125825

[21] Ren S, Tao Y, Jia LU, Cheng P, Zhang J, Zhang H. Fever and arthralgia as the initial symptoms of primary bone marrow diffuse large B-cell lymphoma: a case report. Oncol Lett. 2016;11(5):3428–32. doi:10.3892/ol.2016.4405; 27123129 PMC4840859

[22] Zhang W, Yang H, Sun Y. Primary bone marrow diffuse large B-cell lymphoma: one case report and literature review. J Jilin Univ. 2016;42(4):817–20.

[23] Kim MS, Cho YU, Jang S, Seo EJ, Lee JH, Park CJ. A case of primary bone marrow diffuse large B-cell lymphoma presenting with fibrillar projections and hemophagocytic lymphohistiocytosis. Ann Lab Med. 2017;37(6):544–6. doi:10.3343/alm.2017.37.6.544; 28840996 PMC5587831

[24] Wang W, Zhou GY, Zhang W. Early relapse in a case of primary bone marrow diffuse large B-cell lymphoma treated with rituximab-CHOP. Immunotherapy. 2017;9(5):379–85. doi:10.2217/imt-2017-0005; 28357915

[25] Shea L, Zhao Y, Reddy V, Yacoubian T, Mehta A. Primary bone marrow diffuse large B-cell lymphoma presenting as transverse myelitis. Am J Med Sci. 2018;356(6):561–6. doi:10.1016/j.amjms.2018.09.009; 30447708

[26] Nishida H, Suzuki H, Hori M, Obara K. Primary isolated bone marrow diffuse large B cell lymphoma with long-term complete remission. Leuk Res Rep. 2018;10(2):11–5. doi:10.1016/j.lrr.2018.05.004; 30596009 PMC6308018

[27] Wu Y, Liu J, Cui G, Wang Y, Ai L, Zhang XP. Primary bone marrow diffuse large B-cell lymphoma: report of three cases and literature review. J Leuk Lymphoma. 2018;27(11):678–80.

[28] Guo SP, Jin X, Chen ZL, Jiang HF. Primary bone marrow lymphoma: a case and literature review. Zhejiang J Integr Tradit Chin West Med. 2019;29(3):222–3.

[29] Reed A, Sommerhalder D. The use of R-hyper-CVAD in a rare case of primary bone marrow diffuse large B-cell lymphoma. J Hematol. 2019;8(4):165–7. doi:10.14740/jh559; 32300465 PMC7155813

[30] Chen PT, Jorsan K, Avezbakiyev B, Akhtar C, Wang JC. Aggressive diffuse intermediate size B-cell lymphoma with P53 mutation presented as primary bone marrow lymphoma. J Investig Med High Impact Case Rep. 2020;8:2324709620982765. doi:10.1177/2324709620982765; 33349058 PMC7758647

[31] Tian C, Chen Z, Li Y. Chidamide combined with ibrutinib improved the prognosis of primary bone marrow diffuse large B cell lymphoma. J Int Med Res. 2020;48(7):300060520936053. doi:10.1177/0300060520936053; 32643971 PMC7350052

[32] Kimbrough EO, Jiang L, Parent EE, Bourgeois K, Alhaj Moustafa M, Tun HW, et al. Primary bone marrow lymphoma: de novo and transformed subtypes. J Blood Med. 2022;13:663–71. doi:10.2147/jbm.s384983; 36405430 PMC9673799

[33] Yang CF, Hsiao LT, Chang HY, Hsu CY. Large B-cell lymphoma presenting primarily in bone marrow is frequently associated with haemophagocytic lymphohistiocytosis and has distinct cytogenetic features. Pathology. 2020;52(5):561–7. doi:10.1016/j.pathol.2020.04.005; 32561209

[34] Guo YY, Zhang JY, Sun JF, Nie P, Gao H. Synthesis and application of small molecules approved for the treatment of lymphoma. Eur J Med Chem. 2023;261:115835. doi:10.1016/j.ejmech.2023.115835; 37801827

[35] Nastoupil LJ, Bartlett NL. Navigating the evolving treatment landscape of diffuse large B-cell lymphoma. J Clin Oncol. 2023;41(4):903–13. doi:10.1200/jco.22.01848; 36508700

[36] Sehn LH, Martelli M, Trněný M, Liu W, Bolen CR, Knapp A, et al. A randomized, open-label, Phase III study of obinutuzumab or rituximab plus CHOP in patients with previously untreated diffuse large B-Cell lymphoma: final analysis of GOYA. J Hematol Oncol. 2020;13(1):71. doi:10.1186/s13045-020-00900-7; 32505213 PMC7276080

[37] Ennishi D, Hsi ED, Steidl C, Scott DW. Toward a new molecular taxonomy of diffuse large B-cell lymphoma. Cancer Discov. 2020;10(9):1267–81. doi:10.1158/2159-8290.cd-20-0174; 32616477

[38] Stegemann M, Denker S, Schmitt CA. DLBCL 1L-what to expect beyond R-CHOP? Cancers. 2022;14(6):1453. doi:10.3390/cancers14061453; 35326604 PMC8946010

[39] Johnson PWM, Balasubramanian S, Hodkinson B, Shreeve SM, Sun S, Srinivasan S, et al. Clinical impact of ibrutinib plus R-CHOP in untreated DLBCL coexpressing BCL2 and MYC in the phase 3 PHOENIX trial. Blood Adv. 2023;7(10):2008–17. doi:10.1182/bloodadvances.2022009389; 36696540 PMC10188634

[40] Caimi PF, Ai W, Alderuccio JP, Ardeshna KM, Hamadani M, Hess B, et al. Loncastuximab tesirine in relapsed or refractory diffuse large B-cell lymphoma (LOTIS-2): a multicentre, open-label, single-arm, phase 2 trial. Lancet Oncol. 2021;22(6):790–800. doi:10.1016/s1470-2045(21)00139-x; 33989558

[41] Caimi PF, Ai WZ, Alderuccio JP, Ardeshna KM, Hamadani M, Hess B, et al. Loncastuximab tesirine in relapsed/refractory diffuse large B-cell lymphoma: long-term efficacy and safety from the phase II LOTIS-2 study. Haematologica. 2024;109(4):1184–93. doi:10.3324/haematol.2023.283459; 37646659 PMC10985439

[42] Xu B. Loncastuximab tesirine: an effective therapy for relapsed or refractory diffuse large B-cell lymphoma. Eur J Clin Pharmacol. 2022;78(5):707–19. doi:10.1007/s00228-021-03253-3; 35061047

[43] Tilly H, Morschhauser F, Sehn LH, Friedberg JW, Trněný M, Sharman JP, et al. Polatuzumab vedotin in previously untreated diffuse large B-cell lymphoma. N Engl J Med. 2022;386(4):351–63. doi:10.1056/nejmoa2115304; 34904799 PMC11702892

[44] Nowakowski GS, Chiappella A, Gascoyne RD, Scott DW, Zhang Q, Jurczak W, et al. ROBUST: a phase III study of lenalidomide plus R-CHOP versus placebo plus R-CHOP in Previously untreated patients with ABC-type diffuse large B-cell lymphoma. J Clin Oncol. 2021;39(12):1317–28. doi:10.2217/fon-2016-0130; 33621109 PMC8078325

[45] Nowakowski GS, Hong F, Scott DW, Macon WR, King RL, Habermann TM, et al. Addition of lenalidomide to R-CHOP improves outcomes in newly diagnosed diffuse large B-cell lymphoma in a randomized phase II US intergroup study ECOG-ACRIN E1412. J Clin Oncol. 2021;39(12):1329–38. doi:10.1200/jco.20.01375; 33555941 PMC8078264

[46] Naman J, Shah N, Heyman BM. Antibody therapy for patients with lymphoid malignancies: past and present. Int J Mol Sci. 2025;26(4):1711. doi:10.3390/ijms26041711; 40004173 PMC11855020

